# Changes in transcriptomic response to salinity stress induce the brackish water adaptation in a freshwater snail

**DOI:** 10.1038/s41598-020-73000-8

**Published:** 2020-09-29

**Authors:** Takumi Yokomizo, Yuma Takahashi

**Affiliations:** 1 Graduate School of Science and Engineering, Chiba University, Chiba, Japan; 2 Graduate School of Science, Chiba University, Chiba, Japan

**Keywords:** Ecological genetics, Molecular ecology, Evolutionary genetics, Population genetics, Ecology, Evolution

## Abstract

Studying the mechanisms of the establishment of a population in a novel environment allows us to examine the process of local adaptations and subsequent range expansion. In a river system, detecting genetic or phenotypic differences between a freshwater and brackish water population could contribute to our understanding of the initial process of brackish water adaptation. Here, we investigated behavioural and gene expression responses to salt water in a freshwater and brackish water population of the freshwater snail, *Semisulcospira reiniana*. Although the individuals in brackish water exhibited significantly higher activity in saltwater than freshwater individuals just after sampling, the activity of freshwater individuals had increased in the second observation after rearing, suggesting that their salinity tolerance was plastic rather than genetic. We found 476 and 1002 differentially expressed genes across salinity conditions in the freshwater and brackish water populations, respectively. The major biological process involved in the salinity response of the freshwater population was the biosynthesis and metabolic processing of nitrogen-containing compounds, but that of the brackish water population was influenced by the chitin metabolic process. These results suggest that phenotypic plasticity induces adaptation to brackish water in the freshwater snail by modifying its physiological response to salinity.

## Introduction

Studies concerning distribution patterns of species or populations along the environmental continuum play a significant role in discovering evidence of environmental adaptations. When a species encounters a novel environment during range expansion, adaptation to the environment could aid in the establishment of a population. Adaptations are achieved via two different processes: adaptive evolution and phenotypic plasticity^1,2^. For instance, invasive species, which are introduced into non-native regions by human activity, have been studied extensively with respect to the ongoing process of their adaptive evolution, as they have acquired various traits that are suitable for a novel environment through rapid evolution^3–5^. Evolutionary adaptations, of course, contribute to colonization and expansion into a novel environment in native species^6–8^. Phenotypic plasticity could enhance adaptation to a novel environment, as well as range expansion, because it should enable a species to cope with unfamiliar conditions^9–11^. Both theoretical and empirical studies suggest that individuals with high levels of phenotypic plasticity dominate the edges of distribution ranges^12,13^. For example, plasticity in thermal tolerance is an important trait facilitating survival at the range margins for the fruit fly *Ceratitis capitata*^14^. In general, plasticity makes a significant contribution in adaptation to heterogeneous environments at small spatial scales, or to rapid environmental changes^15,16^. Adaptive plasticity is hypothesized to provide a rapid response to the environment, and also to precede and facilitate adaptive evolution^17–21^. Therefore, understanding the process of both adaptive evolution and phenotypic plasticity is important for revealing the mechanism of range expansion.

In systems in which different environmental conditions are adjacent to each other at relatively small spatial scales, individuals have opportunities to colonize new habitats outside of their current distribution, and thus range expansion can be achieved if a species or population adapts to the environment. Therefore, we can study the ongoing process of adaptation by examining the evolutionary or plastic change in traits along an environmental gradient. A river is one such system, because a steep gradient of salt concentration, which is a significant environmental factor influencing the distribution range of many aquatic species^22–24^, is observed near the estuary due to saltwater intrusion. In freshwater molluscs, tolerance to salinity is critical to populations that reside near estuaries, because salinity affects the osmotic regulation of individuals, and is also involved in their survival, growth, and reproduction^25^. Therefore, tolerance to salinity can assist freshwater species in successfully inhabiting brackish water areas.

Detecting genetic or phenotypic variation in individuals living in freshwater or brackish water will contribute to our understanding of the mechanisms of adaptation to brackish water. Several studies have evaluated differences in tolerance to salinity between freshwater and brackish water species^26,27^. However, interspecific comparisons, in general, may not clarify the initial process of adaptation to brackish water. The differences between species include differences that are responsible for adaptation to brackish water, those that accumulated after the adaptation, and those which are totally unrelated to the adaptation to saltwater. Conversely, differences within species include only the differences responsible for the adaptation to brackish water, because few mutations accumulate in the time following the isolation of populations. Thus, intraspecific comparisons, such as comparisons between a freshwater and a brackish water population within a single species, enable us to clarify the initial process of adaptation to brackish water, and the mechanisms that lead to the expansion of species distributions into brackish water.

The freshwater snail, *Semisulcospira reiniana*, inhabits a broad range of river environments. Although the snail primarily occupies freshwater areas, it also lives in brackish water areas in several rivers of Japan. Here, we investigate the behavioural and gene expression responses to saltwater in adult individuals of the species. First, to examine their activity under salinity stress, we compared activity levels in saltwater using individuals in a freshwater population and its adjacent brackish water population. Second, transcriptome analysis was conducted to find gene(s) differentially responding to saltwater between the populations. Using transcriptome data, we also assessed the genetic distance between the two adjacent populations.

## Results

### Genetic differentiation

We obtained 983,061 transcripts with a mean length of 556 bp via de novo assembly. Among the 978 metazoan core gene orthologues, 969 genes (99.1%) were identified completely. We ran the CD-HIT program using 164,656 putative coding sequences (CDS), which provided 94,676 clustered CDS transcripts. Using these CDS transcripts, we identified 5871 SNPs on the contigs annotated against all protein sequences of *Crassostrea gigas* by BLAST search. We used these SNPs for the following population genetic analysis.

The BayeScan program did not identify any outlier loci, indicating that all SNPs were selectively neutral between the two populations. Using all 5871 SNPs, the *F*_ST_ value between the freshwater and brackish water populations was estimated at 0.011.

### Behavioural response to saltwater

**Figure 1:**
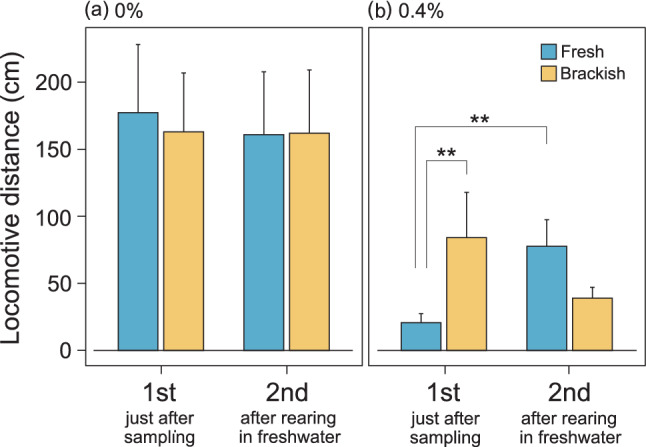
Changes in the locomotive distance of individuals collected in freshwater and brackish water area with observation in 0% (**a**) and 0.4% saltwater (**b**). The first observation was conducted just after sampling and the second was conducted after rearing in freshwater. Error bars are SEM. **P < 0.01 in post hoc comparison (α = 0.0125).

The locomotive activity in 0% saltwater was not significantly different between populations, either in the first observation immediately after collecting, or in the second observation after rearing in experimental freshwater for one week following the first observation (population [P]: χ^2^ = 0.02, *P* = 0.89; the number of days after collecting [D]: χ^2^ = 0.03, *P* = 0.85; P × D: χ^2^ = 0.03, *P* = 0.87, Fig. 1a). In contrast, we found interaction effects for the locomotive activity in 0.4% saltwater (P: χ^2^ = 1.06, *P* = 0.30; D: χ^2^ = 0.64, *P* = 0.42; P × D: χ^2^ = 11.3, *P* < 0.001, Fig. 1b), indicating a differential response to the experience of being reared in an experimental freshwater setup for one week between populations. Post hoc comparison found significant differences in locomotive activity between populations on the first observation, immediately after sampling. The locomotive activity of the freshwater population in 0.4% saltwater was higher during the second observation, after rearing in freshwater. Although we were not able to identify a statistically significant difference, the locomotive activity of the brackish water population in 0.4% saltwater tended to decrease between the first observation and the second observation. Consequently, no significant difference between populations in locomotive activity in 0.4% saltwater was found at the second behavioural observation.

### Gene expression response to salinity

All freshwater and brackish water individuals were active in 0% saltwater. In 0.6% saltwater, all brackish water individuals were active, while three of the four freshwater individuals were not. In 0.8% saltwater, two of the four brackish water individuals were active, but all the freshwater individuals withdrew into their shells to avoid the salinity. These results supported the hypothesis that brackish water individuals have a higher tolerance to salinity, as shown previously.

Among the164,656 putative CDS, 81,495 (49.5%) of them were annotated against all protein sequences of *C. gigas*, using BLAST search. These annotated sequences comprised 34,816 genes. We used these genes for gene expression analysis.

**Figure 2:**
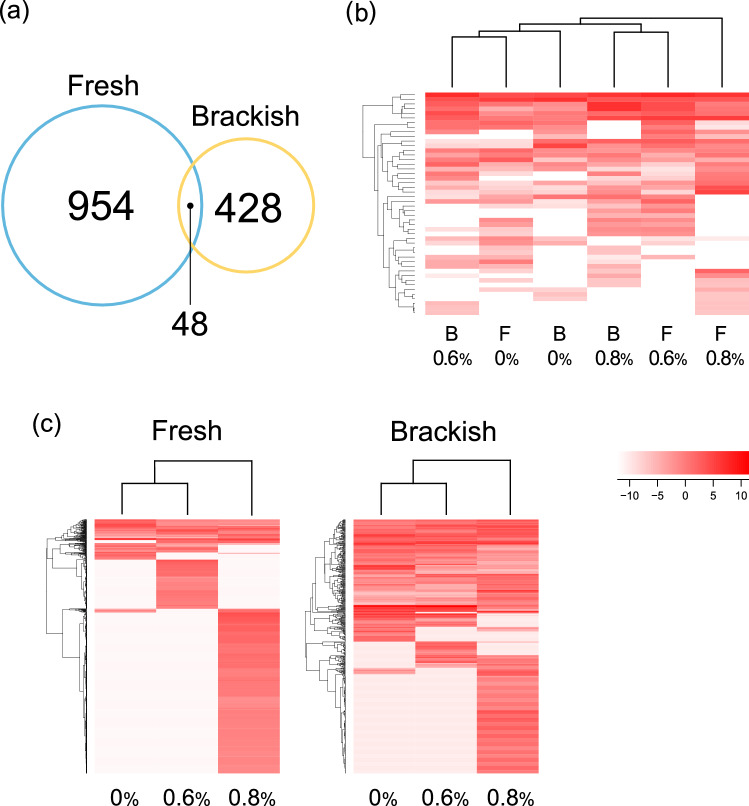
The number of differentially expressed genes (DEGs) and their expression patterns. (**a**) The number of DEGs of the freshwater and brackish water populations. (**b**,**c**) The expression patterns of DEGs shared between the two populations (**b**) and all DEGs in each population (**c**). Colour scale represents the log scaled value of mean FPKM of three individuals in each salt concentration.

The number of differentially expressed genes (DEGs) among the three salt concentrations was higher in the freshwater population (1002) than in the brackish population (476), indicating that many more genes were differentially expressed in the freshwater population than in the brackish water population (Fig. 2a; Supplementary Tables S1 and S2). Among these DEGs, 48 genes were shared between the two populations, but the expression patterns of the shared DEGs differed from each other (Fig. 2b; Supplementary Table S3). The number of genes highly expressed in 0.8% saltwater was higher than that expressed in other saltwater concentrations, irrespective of the population, probably due to strong salinity stress (Fig. 2c).

The GO terms that were significantly enriched within the set of DEGs in the freshwater and brackish water populations are summarized in Supplementary Tables S4 and S5, respectively. We found 62 terms in the freshwater population and 53 terms in the brackish one. In the freshwater population, 45 terms were unique to the population (Table 1), including those involved in the biosynthesis and metabolic processes of nitrogen-containing compounds. In the brackish water population, 36 terms were unique to the population (Table 2), including those involved in the chitin metabolic process and urea transport.

**Table 1:** GO terms enriched only in the freshwater population.

GO_ID	Term	Category	FDR
GO:1901566	Organonitrogen compound biosynthetic process	BP	1.5 × 10^−8^
GO:0019538	Protein metabolic process	BP	8.4 × 10^−7^
GO:0032991	Protein-containing complex	CC	7.1 × 10^−6^
GO:0043229	Intracellular organelle	CC	1.1 × 10^−5^
GO:0044271	Cellular nitrogen compound biosynthetic process	BP	1.1 × 10^−5^
GO:0003723	RNA binding	MF	1.5 × 10^−5^
GO:0043226	Organelle	CC	1.9 × 10^−5^
GO:0005622	Intracellular	CC	2.1 × 10^−5^
GO:0044424	Intracellular part	CC	2.1 × 10^−5^
GO:0034645	Cellular macromolecule biosynthetic process	BP	2.4 × 10^−5^
GO:0009059	Macromolecule biosynthetic process	BP	3 × 10^−5^
GO:0004190	Aspartic-type endopeptidase activity	MF	6.9 × 10^−5^
GO:0070001	Aspartic-type peptidase activity	MF	6.9 × 10^−5^
GO:0010467	Gene expression	BP	8.7 × 10^−5^
GO:0051082	Unfolded protein binding	MF	0.00012
GO:0044444	Cytoplasmic part	CC	0.00013
GO:0034641	Cellular nitrogen compound metabolic process	BP	0.00018
GO:0044249	Cellular biosynthetic process	BP	0.00028
GO:0005737	Cytoplasm	CC	0.00036
GO:0006457	Protein folding	BP	0.00045
GO:0006807	Nitrogen compound metabolic process	BP	0.00051
GO:0044238	Primary metabolic process	BP	0.00059
GO:1901576	Organic substance biosynthetic process	BP	0.00109
GO:0005623	Cell	CC	0.00111
GO:0044464	Cell part	CC	0.00118
GO:0071704	Organic substance metabolic process	BP	0.00259
GO:0009058	Biosynthetic process	BP	0.00337
GO:0009987	Cellular process	BP	0.0038
GO:0043170	Macromolecule metabolic process	BP	0.00472
GO:0044267	Cellular protein metabolic process	BP	0.00902
GO:0005856	Cytoskeleton	CC	0.01479
GO:0090079	Translation regulator activity, nucleic acid binding	MF	0.01554
GO:0008135	Translation factor activity, RNA binding	MF	0.01554
GO:0006165	Nucleoside diphosphate phosphorylation	BP	0.01919
GO:0046939	Nucleotide phosphorylation	BP	0.01919
GO:0072521	Purine-containing compound metabolic process	BP	0.02035
GO:0016864	Intramolecular oxidoreductase activity, transposing S–S bonds	MF	0.0277
GO:0003756	Protein disulfide isomerase activity	MF	0.0277
GO:0045182	Translation regulator activity	MF	0.02875
GO:0009132	Nucleoside diphosphate metabolic process	BP	0.02891
GO:0045275	Respiratory chain complex III	CC	0.02891
GO:0006122	Mitochondrial electron transport, ubiquinol to cytochrome c	BP	0.02891
GO:0046034	ATP metabolic process	BP	0.02931
GO:0009116	Nucleoside metabolic process	BP	0.04158
GO:1901657	Glycosyl compound metabolic process	BP	0.04872

Full list of GO terms enriched is shown in Supplementary Table S4.

*BP* biological process, *MF* molecular function, *CC* cellular component.

**Table 2:** GO terms enriched only in the brackish water population.

GO_ID	Term	Category	FDR
GO:0008061	Chitin binding	MF	2.43 × 10^−6^
GO:0006022	Aminoglycan metabolic process	BP	2.43 × 10^−6^
GO:0006030	Chitin metabolic process	BP	3.14 × 10^−5^
GO:1901071	Glucosamine-containing compound metabolic process	BP	4.40 × 10^−5^
GO:0006040	Amino sugar metabolic process	BP	7.25 × 10^−5^
GO:0070011	Peptidase activity, acting on l-amino acid peptides	MF	0.00055
GO:0008233	Peptidase activity	MF	0.00062
GO:0006508	Proteolysis	BP	0.00248
GO:0004866	Endopeptidase inhibitor activity	MF	0.003
GO:0061135	Endopeptidase regulator activity	MF	0.003
GO:0042807	Central vacuole	CC	0.00733
GO:0009705	Plant-type vacuole membrane	CC	0.00733
GO:0080170	Hydrogen peroxide transmembrane transport	BP	0.00733
GO:0030414	Peptidase inhibitor activity	MF	0.00921
GO:0061134	Peptidase regulator activity	MF	0.00952
GO:0005372	Water transmembrane transporter activity	MF	0.01089
GO:0042044	Fluid transport	BP	0.01089
GO:0000326	Protein storage vacuole	CC	0.01089
GO:0000325	Plant-type vacuole	CC	0.01089
GO:0015250	Water channel activity	MF	0.01089
GO:0006833	Water transport	BP	0.01089
GO:0003746	Translation elongation factor activity	MF	0.01089
GO:0009941	Chloroplast envelope	CC	0.01533
GO:0019755	One-carbon compound transport	BP	0.01617
GO:0015204	Urea transmembrane transporter activity	MF	0.01617
GO:0004857	Enzyme inhibitor activity	MF	0.01617
GO:0071918	Urea transmembrane transport	BP	0.01617
GO:0015840	Urea transport	BP	0.01617
GO:0009526	Plastid envelope	CC	0.01678
GO:0005328	Neurotransmitter:sodium symporter activity	MF	0.03441
GO:0051181	Cofactor transport	BP	0.03441
GO:0005576	Extracellular region	CC	0.03541
GO:0004867	Serine-type endopeptidase inhibitor activity	MF	0.03541
GO:0015370	Solute:sodium symporter activity	MF	0.03645
GO:0052717	tRNA-specific adenosine-34 deaminase activity	MF	0.04342
GO:0005326	Neurotransmitter transporter activity	MF	0.04406

Full list of GO terms enriched is shown in Supplementary Table S5.

*BP* biological process, *MF* molecular function, *CC* cellular component.

## Discussion

Either adaptive evolution or phenotypic plasticity, or both, can contribute to the establishment of a population in a novel environment^1,2^. In a river, salinity gradually increases toward the estuary, creating a steep environmental gradient. Freshwater organisms typically do not possess the physiological mechanisms to cope with high salinity. The distribution of freshwater species is limited to a specific range between brackish and freshwater areas. Therefore, range expansion to brackish areas requires tolerance to salinity, and thus brackish water populations display different salinity responses than freshwater populations. In the present study, we confirmed that individuals from a population living in brackish water displayed different salinity responses from those of a freshwater population, in terms of both activity and physiology. Our findings could clarify the understanding of the mechanisms of brackish water adaptations via adaptive evolution and/or phenotypic plasticity.

The *F*_ST_ value between two populations, which we observed, is very low (0.011) compared with previous studies in snails^28,29^. This value of *F*_ST_ is considered to indicate that the two adjacent populations exhibit little genetic differentiation^30^, supporting our assumption. The brackish water population is suggested to have colonized their novel environment recently or regularly receive immigrants from the freshwater population. The brackish water population appears to be in the initial stages of adaptation to brackish water, and thus few mutations have accumulated in the population. Therefore, in our study system, we could observe a phenomenon that occurs in the early stages of brackish water adaptation.

In 0.4% saltwater, we found significantly higher activity levels in individuals in brackish water than in individuals in fresh water immediately after transfer from one environment to the other, indicating that individuals in brackish water can be active even in conditions of salinity. The lack of significant differences between the activity levels of populations at the second observation is probably due to acclimation to the same rearing environment for one week. The increased activity of freshwater individuals at the second observation may be due to their acclimation to unexpected changes in water conditions, because the 0% saltwater used for keeping individuals may be slightly hypertonic due to the effect of their faeces and a crushed oyster shell. The lack of difference between the activity levels of populations in 0.4% saltwater after one week suggests that individuals have the ability to change their activity level according to water conditions. If higher activity in saltwater is derived from high tolerance to salinity, our results suggest that phenotypic plasticity may contribute to tolerance to salinity in individuals in brackish water. The hypothesis that phenotypic plasticity may contribute to salinity tolerance is supported by the absence of outlier loci in the two populations, which showed distinct responses to salinity. However, we cannot rule out adaptive evolution caused by genetic differences, especially in non-coding regions.

The relationship between phenotypic plasticity and adaptation to novel environments has been long studied. Baldwin^17^ suggested that adaptive plasticity plays an important role in the establishment and persistence of populations in new environments. Also, an adaptive phenotype initially accomplished by plasticity is hypothesized to sometimes become genetically-encoded, so that establishment in a new environment is achieved^18,19^. Our results indicate that *S. reiniana* can acclimate to salinity even in individuals grown in fresh water. Individuals in brackish water may cope with salinity via phenotypic plasticity, and may be in the process of adaptation to a brackish environment as suggested by Baldwin’s hypothesis.

In our transcriptome analysis, we found that the expression level of 48 genes changed with salinity in the two populations, suggesting that these genes could be involved in the basis of saltwater response in *S. reiniana*. We detected four genes encoding proteins with von Willebrand factor domains within the shared DEG set. Von Willebrand factor, which plays a key role in normal hemostasis, is suggested to be expressed when extracellular NaCl levels are elevated^31^. High salinity would have a deleterious effect on freshwater snails, by promoting excess thrombus formation. In the freshwater population we found several genes involved in the biosynthesis and metabolic processing of nitrogen-containing compounds, such as glutamate synthase and glutamine synthetase. Gene ontology analysis also revealed DEGs enriched in nitrogen compound biosynthetic and metabolic processes. Nitrogen-containing compounds accumulate in plant species subjected to salinity stress and are involved in osmoregulation^32^. Nitrogen metabolism is also involved in the salinity responses of many molluscan species^33^, indicating that individuals in freshwater have common processes in response to salinity. A different pattern of gene expression was observed in the brackish water population, in which we noted that several genes associated with chitin metabolism were differentially expressed, including chitin synthase C and chitotriosidase-1. While chitin is an essential component of mollusc shells, its function in osmoregulation has not been reported. However, Lv et al.^34^ suggested that chitinase is associated with the response to salinity in crustacea, leading to the hypothesis that a similar process occurs in *S. reiniana*. DEGs in the brackish water population were enriched not only in chitin metabolism but also in urea transport. Urea functions as an osmolyte, and holds special importance for cell volume preservation in an aquatic snail under hyperosmotic stress^35^. However, it is not clear why genes associated with urea were enriched only in the brackish water population and those associated with nitrogen-containing compounds only in the freshwater population; although both of them are osmolytes which act to prevent water loss in hyperosmotic environments. In summary, more than half of the GO terms that were enriched within each DEG set did not overlap, indicating that the biological processes in response to salinity were different in the two populations. These differences in gene expression patterns may be caused by plasticity in brackish water adaptations. Freshwater individuals would acclimate to saltwater in a few days, and individuals in both populations would exhibit similar gene expression patterns.

Our results indicate that individuals in fresh water and brackish water show different responses to salinity with respect to activity levels and gene expression patterns. The establishment of a population in a brackish water area requires the activation of biological processes that can cope with high salinity. Given the results of our behavioural and transcriptome analyses, metabolic processing of chitin and urea may lead to higher activity in saltwater in individuals from brackish water. However, our behavioural observations suggest that individuals from fresh water may have enhanced activity under salinity stress. In summary, acclimation to hypertonic conditions and alteration of salinity responses via physiological processes can contribute to the early stage of brackish water adaptation in freshwater snails.

Note that our results do not explain why range expansion to a brackish water environment has not been achieved in all rivers, despite their potential for acclimation to salinity. Evolutionary differences in the strength of plasticity and long-term salinity tolerance may be associated with the success of brackish water adaptations. Further investigation into the effects of long-term exposure to salinity on the survival, growth, and reproduction of individuals in fresh and brackish water is required.

## Materials and methods

### Species and sampling

*Semisulcospira reiniana* is a common freshwater snail in Japan and mainly inhabits the freshwater areas of rivers. We collected adult individuals of *S. reiniana* from freshwater (35° 15′ 15″ N, 136° 41′ 09″ E) and brackish water (35° 07′ 22″ N, 136° 41′ 27″ E) areas of Kiso river in Japan, in May 2018 and June 2019. While water level fluctuations caused by the tidal cycles occur at both sampling sites, seawater does not reach the freshwater site. The salt concentration of the freshwater site was almost 0%, while the salt concentration at the brackish water site fluctuates from 0 to 0.5% according to the tidal cycle. Since these two populations are located relatively close to each other (15 km apart), we assume that the two populations have not diverged genetically. The snail specimens were preserved in a container (13 × 13 × 20 cm) maintained at 23 °C for one day until the onset of experiments.

### Behavioural response to salinity

Individuals collected in freshwater and brackish water populations were examined for activity under concentrations of 0% and 0.4% saltwater. Ten individuals were tested in each salinity condition for each of these two populations (total sample size was 40). Snails were put separately in individual bowls (φ13 × 3.5 cm) filled with 0% or 0.4% saltwater, prepared from decalcified tap water. The bowls were kept under laboratory conditions at a temperature of approximately 23 °C. Thirty minutes after putting the individuals into the bowls, they were monitored by a camera (400-CAM061, Sanwa Direct) for two hours to quantify locomotive activity. Images were taken every 30 s and used to create time-lapse movies. We tracked the position of each individual using the tracking software UMATracker^36^. Using the snail trajectories, the total locomotion distance for two hours was calculated for each individual. After the first behavioural observation, we put individuals back into the containers filled with fresh water (decalcified tap water). We added a crushed oyster shell to the containers to keep the water clear. One week later, we conducted the second behavioural observation following the same procedure, and using the same sample set.

### Gene expression response to salinity

Three freshwater and three brackish water individuals, reared under the freshwater condition for one week after collection, were exposed to 0%, 0.6%, or 0.8% saltwater for three hours in the laboratory. While 0% saltwater was assumed to be a typical optimum environment for *S. reiniana* in the natural population, 0.6% and 0.8% were expected to be high and extremely high salinity conditions in the tidal cycle, respectively. Soon after exposure, we checked to see whether the snails were active. Individuals were dissected and their epidermis was preserved in 750 μl of RNA*later* Stabilization Solution (Invitrogen). The samples were kept at − 80 °C until the extraction of total RNA.

Total RNA was extracted using Maxwell 16 LEV Plant RNA Kit with the Maxwell 16 Research Instrument (Promega) according to the manufacturer’s instructions. Electrophoresis on 1% agarose gels was performed to check for RNA degradation. RNA concentrations were estimated using a Qubit 2.0 Fluorometer (Invitrogen). RNA purity was estimated using a BioSpec-nano (Shimadzu). The cDNA library was constructed using TruSeq RNA Sample Prep Kits. Paired-end (150 bp) RNA sequencing (RNA-Seq) was performed on the Illumina NovaSeq6000 platform. After the removal of adaptor sequences and low-quality reads using Trimmomatic^37^, we used FastQC (https://www.bioinformatics.babraham.ac.uk/projects/fastqc/) for quality control. The remaining high-quality reads were used for de novo assembly using Trinity^38^.

To estimate gene expression levels, all reads of each sample were mapped to the reference transcripts using RSEM^39^. The read count data was used for gene expression analysis. We searched for homologues of every *S. reiniana* gene using BLAST searches for all protein sequences of *C. gigas*. Genes with the best hit and with an e-value < 0.0001 were used for gene expression analysis. DEGs among the three salt concentrations were detected using the TCC package^40,41^. We considered genes with *q* < 0.05 as DEGs. Gene ontology (GO) enrichment analysis of the DEGs was performed using Blast2GO software^42^.

### ***F***_ST_ calculation

Putative coding regions were extracted from the reference transcripts using TransDecoder (https://github.com/TransDecoder/TransDecoder/wiki), providing the reference transcripts only contained CDS. They were clustered based on sequence identities of 90% to remove redundancy, using the CD-HIT program^43^. We used STAR for mapping all reads of each sample to the clustered CDS reference. We then used GATK^44^ to identify SNPs. We used the homologues of *C. gigas*, which were detected in the same manner as described in a previous section. Genes with the best hit and with an e-value < 0.0001 were used to estimate the genetic structure of the two populations. To identify outlier loci which were not selectively neutral between the two populations, we ran the BayeScan program^45^ with default parameters. Putatively neutral loci were used to estimate *F*_ST_ between the two populations using Arlequin^46^.

### Statistical analyses

Locomotive distances in 0% and 0.4% saltwater were analyzed by the generalized linear model (GLM) with a Gamma distribution. Population (i.e., freshwater, and brackish water population) and the number of days after collecting (i.e., first and second observation) were included as explanatory variables. Post hoc test for all four pairwise comparisons was conducted using the GLM with a Gamma distribution. Since the analysis was performed four times, we applied the Bonferroni correction to account for multiple comparisons (Bonferroni-adjusted α was 0.0125). All statistical analyses were performed in R 3.4.2.

## Supplementary information

Supplementary Table S1.

Supplementary Table S2.

Supplementary Table S3.

Supplementary Table S4.

Supplementary Table S5.

## Data Availability

All raw transcriptome read data were deposited in the DDBJ Sequenced Read Archive under accession numbers SAMD00218387–SAMD00218404. Behavioural and transcriptome data are available in the Dryad Data Archive at https://doi.org/10.5061/dryad.jdfn2z37w.

## References

[1] Gienapp P, Teplitsky C, Alho JS, Mills JA, Merilä J (2008). Climate change and evolution: disentangling environmental and genetic responses. Mol. Ecol..

[2] Anderson JT, Inouye DW, McKinney AM, Colautti RI, Mitchell-Olds T (2012). Phenotypic plasticity and adaptive evolution contribute to advancing flowering phenology in response to climate change. Proc. R. Soc. B Biol. Sci..

[3] Siemann E, Rogers WE (2001). Genetic differences in growth of an invasive tree species. Ecol. Lett..

[4] Bossdorf O, Prati D, Auge H, Schmid B (2004). Reduced competitive ability in an invasive plant. Ecol. Lett..

[5] Maron JL, Vilà M, Bommarco R, Elmendorf S, Beardsley P (2004). Rapid evolution of an invasive plant. Ecol. Monogr..

[6] Byrne K, Nichols RA (1999). *Culex pipiens* in London underground tunnels: differentiation between surface and subterranean populations. Heredity.

[7] Lee CE (1999). Rapid and repeated invasions of fresh water by the copepod *Eurytemora affinis*. Evolution.

[8] Linnen CR (2013). Adaptive evolution of multiple traits through multiple mutations at a single gene. Science.

[9] Yeh PJ, Price TD (2004). Adaptive phenotypic plasticity and the successful colonization of a novel environment. Am. Nat..

[10] Price TD, Yeh PJ, Harr B (2008). Phenotypic plasticity and the evolution of a socially selected trait following colonization of a novel environment. Am. Nat..

[11] Lande R (2015). Evolution of phenotypic plasticity in colonizing species. Mol. Ecol..

[12] Chevin LM, Lande R (2011). Adaptation to marginal habitats by evolution of increased phenotypic plasticity. J. Evol. Biol..

[13] Orizaola G, Laurila A (2016). Developmental plasticity increases at the northern range margin in a warm-dependent amphibian. Evol. Appl..

[14] Nyamukondiwa C, Kleynhans E, Terblanche JS (2010). Phenotypic plasticity of thermal tolerance contributes to the invasion potential of Mediterranean fruit flies (*Ceratitis capitata*). Ecol. Entomol..

[15] Richards CL, Bossdorf O, Muth NZ, Gurevitch J, Pigliucci M (2006). Jack of all trades, master of some? On the role of phenotypic plasticity in plant invasions. Ecol. Lett..

[16] Crispo E (2008). Modifying effects of phenotypic plasticity on interactions among natural selection, adaptation and gene flow. J. Evol. Biol..

[17] Baldwin JM (1896). A new factor in evolution. Am. Nat..

[18] Waddington CH (1961). Genetic assimilation. Adv. Genet..

[19] Price TD, Qvarnström A, Irwin DE (2003). The role of phenotypic plasticity in driving genetic evolution. Proc. R. Soc. B Biol. Sci..

[20] Lande R (2009). Adaptation to an extraordinary environment by evolution of phenotypic plasticity and genetic assimilation. J. Evol. Biol..

[21] Levis NA, Pfennig DW (2016). Evaluating ‘Plasticity-First’ evolution in nature: key criteria and empirical approaches. Trends Ecol. Evol..

[22] Charmantier G (1998). Ontogeny of osmoregulation in crustaceans: a review. Invertebr. Reprod. Dev..

[23] Cervetto G, Gaudy R, Pagano M (1999). Influence of salinity on the distribution of *Acartia tonsa* (Copepoda, Calanoida). J. Exp. Mar. Bio. Ecol..

[24] Ho P-T (2019). Impacts of salt stress on locomotor and transcriptomic responses in the intertidal gastropod *Batillaria attramentaria*. Biol. Bull..

[25] Yang S (2018). The salinity tolerance of the invasive golden apple snail (*Pomacea canaliculata*). Molluscan Res..

[26] Deaton LE, Derby JGS, Subhedar N, Greenberg MJ (1989). Osmoregulation and salinity tolerance in two species of bivalve mollusc: *Limnoperna fortunei* and *Mytilopsis leucophaeta*. J. Exp. Mar. Bio. Ecol..

[27] Jordan PJ, Deaton LE (1999). Osmotic regulation and salinity tolerance in the freshwater snail *Pomacea bridgesi* and the freshwater clam *Lampsilis teres*. Comp. Biochem. Physiol. A Mol. Integr. Physiol..

[28] Bouétard A, Côte J, Besnard AL, Collinet M, Coutellec MA (2014). Environmental versus anthropogenic effects on population adaptive divergence in the freshwater snail *Lymnaea stagnalis*. PLoS ONE.

[29] Sinclair CS (2010). Surfing snails: population genetics of the land snail *Ventridens ligera* (Stylommatophora: Zonitidae) in the Potomac Gorge. Am. Malacol. Bull..

[30] Hartl DL, Clark AG (2007). Principles of Population Genetics.

[31] Dmitrieva NI, Burg MB (2013). Elevation of extracellular NaCl increases secretion of von Willebrand Factor from endothelial cells. FASEB J..

[32] Mansour MMF (2000). Nitrogen containing compounds and adaptation of plants to salinity stress. Biol. Plant..

[33] Somero, G. N. & Bowlus, R. D. Osmolytes and metabolic end products of molluscs: the design of compatible solute systems. in *Mollusca*, Vol. 2. *Environ. Biochem. Physiol.* 77–100 (1983).

[34] Lv J (2013). Transcriptome analysis of *Portunus trituberculatus* in response to salinity stress provides insights into the molecular basis of osmoregulation. PLoS ONE.

[35] Wiesenthal AA, Müller C, Harder K, Hildebrandt JP (2019). Alanine, proline and urea are major organic osmolytes in the snail *Theodoxus fluviatilis* under hyperosmotic stress. J. Exp. Biol..

[36] Yamanaka O, Takeuchi R (2018). UMATracker: an intuitive image-based tracking platform. J. Exp. Biol..

[37] Bolger AM, Lohse M, Usadel B (2014). Trimmomatic: a flexible trimmer for illumina sequence data. Bioinformatics.

[38] Grabherr MG (2011). Full-length transcriptome assembly from RNA-Seq data without a reference genome. Nat. Biotechnol..

[39] Li B, Dewey CN (2011). Assembly of non-unique insertion content using next-generation sequencing. BMC Bioinform..

[40] Sun J, Nishiyama T, Shimizu K, Kadota K (2013). TCC: An R package for comparing tag count data with robust normalization strategies. BMC Bioinform..

[41] Tang M, Sun J, Shimizu K, Kadota K (2015). Evaluation of methods for differential expression analysis on multi-group RNA-seq count data. BMC Bioinform..

[42] Conesa A (2005). Blast2GO: a universal tool for annotation, visualization and analysis in functional genomics research. Bioinformatics.

[43] Li W, Godzik A (2006). Cd-hit: a fast program for clustering and comparing large sets of protein or nucleotide sequences. Bioinformatics.

[44] McKenna A (2010). The genome analysis toolkit: a MapReduce framework for analyzing next-generation DNA sequencing data. Genome Res..

[45] Foll M, Gaggiotti O (2008). A genome-scan method to identify selected loci appropriate for both dominant and codominant markers: a Bayesian perspective. Genetics.

[46] Excoffier L, Lischer HEL (2010). Arlequin suite ver 3.5: a new series of programs to perform population genetics analyses under Linux and Windows. Mol. Ecol. Resour..

